# Memantine as an Augmentation Therapy for Anxiety Disorders

**DOI:** 10.1155/2012/749796

**Published:** 2012-02-08

**Authors:** Thomas L. Schwartz, Umar A. Siddiqui, Shafi Raza

**Affiliations:** Department of Psychiatry, SUNY Upstate Medical University, 750 E. Adams Street, Syracuse, NY 13210, USA

## Abstract

*Objective*. Glutamate, an excitatory neurotransmitter in the central nervous system (CNS), may play a role in the development of anxiety. Memantine partially blocks N-methyl-D-aspartate (NMDA) receptors' glutamate channels located in the CNS. This paper evaluates memantine as an augmentation therapy for treatment of anxiety. *Methods*. 15 consecutive partially responding anxious patients were treated with adjunctive memantine for 10 weeks. Memantine was dosed 5–20 mg/day. *Result*. Memantine augmentation resulted in clinically relevant reduction in anxiety symptoms when compared to baseline. Forty percent of patients achieved remission (HAM-A ≥ 7). Memantine improved sleep quality. Mean dose was 14 mg/d (range 5–20 mg/d). Typical adverse events included nausea and headache. *Conclusion*. The NMDA receptor antagonist memantine may be an effective augmentation therapy in patients with treatment-resistant anxiety.

## 1. Introduction

Anxiety disorders are some of the most occurring forms of psychiatric disorders seen in clinical practice and are associated with a 1-year prevalence rate of 12% in adults [1]. Selective serotonin reuptake inhibitors (SSRIs) are commonly used for the treatment of anxiety disorders; however, complete resolution of symptoms may only be seen in approximately 50% of patients [2]. Augmentation therapy is a common approach to managing patients who remain symptomatic despite adequate treatment with conventional antidepressant anxiolytics [3]. Glutamate, the predominant excitatory neurotransmitter in the central nervous system, is thought to play an important role in the pathophysiology of anxiety [4, 5]. FDA has approved memantine, as a noncompetitive N-methyl-D-aspartate (NMDA) glutamate receptor antagonist for the treatment of Alzheimer's disease [6]. In preclinical reports and other clinical trials, memantine has been shown to be effective in the treatment of obsessive compulsive and posttraumatic disorders but has never been evaluated for generalized or social anxiety disorders [7–9].

Memantine partially inhibits N-methyl-D-aspartate (NMDA) glutamate receptors thus dampening synaptic glutamate availability [10].

The authors feel that memantine, given its ability to manipulate the GABA-glutamate balance by lowering glutamate without major side effects (weight gain, sexual problems, (i.e., SSRI/SNRI), or addiction (i.e., sedatives)), may be a reasonable add-on or augmentation strategy to better alleviate anxiety in SNRI or SSRI partial responders.

### 1.1. Case Presentation

All patients in this paper suffered from either social anxiety disorder (SAD) or generalized anxiety disorder (GAD). All patients were treated previously with adequate doses of approved selective serotonin reuptake inhibitors (SSRIs). However, despite this treatment, all were considered to be partial responders and not felt to be in remission in regards to their anxious symptoms.

All agreed to be enrolled in an open-label case series where memantine was used adjunctively with their preexisting SSRIs in order to further mitigate their anxiety. None of the patients were allowed to take benzodiazepines during the course of the study. Dissociative or psychotic patients were excluded.

## 2. Methods

This was a ten-week, single-center, open-label study for which fifteen patients were enrolled after obtaining Institutional review board approval. Main eligibility criteria included:

- male and female outpatients aged 18–65 with a DSM-IV diagnosis of either generalized or social anxiety disorder based on MINI diagnostic interview;
- rating of at least “moderately ill” (score of 4) on the Clinical Global Impressions of Severity (CGI-S) scale and score of at least 10 on HAM-A and 5 on HADS anxiety subscale;
- failure to respond adequately to trial of conventional anxiolytic;
- adequate treatment duration was considered 4 weeks at therapeutic dose;
- adequate response is defined as an improvement of ≥50% in symptoms of anxiety on current anxiolytic therapy, as measured subjectively by patients or objectively by physician;
- stable on current psychiatric medication for ≥6 weeks prior to study entry.

**Figure 1:**
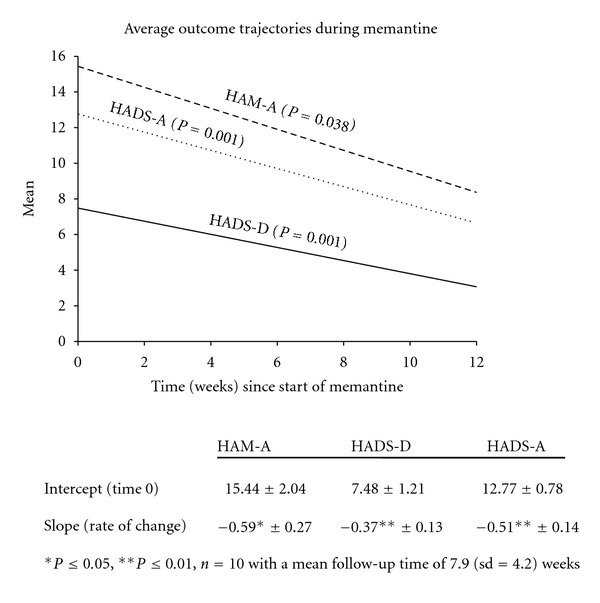
Mean outcome trajectories were assessed by implementing a random intercept model to account for the within-subject dependency due to repeated assessments over a twelve-week study period.

Memantine was initiated at 5 mg/d. It could be flexibly dosed upwards by 5 mg every week based on effectiveness and tolerability to a maximum dose of 20 mg/day. Total daily doses of memantine were divided between morning and evening and taken with food. Effectiveness was assessed using Hamilton Anxiety Rating Scale (HAM-A), Hospital Anxiety and Depression Scale (HADS), CGI of Change (CGI-C), and Pittsburgh Sleep Quality Index (PSQI). Patients with at least one postbaseline efficacy measurement were included in the efficacy dataset and analyzed using the last-observation-carried-forward (LOCF) technique. Comparisons to baseline in HAM-A and HADS were performed. A responder was defined as a patient with a reduction of ≥50% in HAM-A total score. Remission was defined as an HAM-A total score ≤7. Mean outcome trajectories were assessed by implementing a random intercept model to account for the within subject dependency due to assessments over the study period (Figure 1).

## 3. Results

There were 15 patients enrolled for the study, 11 females and 4 males. Mean age was 44 years (range 26–63). All 15 patients (100%) had a diagnosis of generalized anxiety disorder (GAD) while 4 (26%) patients also had comorbid social anxiety disorder, and they all (100%) were using SSRIs at the time of the enrollment with a mean dose of 40 mg/day (Table 1). Nine patients (60%) were mild to moderately ill, while 6 patients (40%) were markedly ill at the baseline visit according to the CGI scale. Of the 15 enrolled patients, 10 were included in the efficacy analysis as each had at least two consecutive visits. Two patients discontinued treatment due to moderate adverse events (nausea and dizziness) after their second study visit. The mean final dose of memantine was approximately 14 mg/day. Five patients withdrew consent. Seven patients completed the trial fully. Memantine augmentation resulted in clinically relevant reduction in anxiety symptom, as shown by significant reductions from baseline in mean HAM-A and HADS-A total score. Mean HAM-A at baseline was 16.6 which lowered to 10.9. Similarly mean HAD-A dropped from baseline value of 13.2 to 8.4. The number of patients considered responders (reduction of ≥50% in HAM-A total score) at endpoint was 6 (60%). The number of patients who achieved remission (HAM-A ≤7) at endpoint was 4 (40%). Memantine also improved sleep quality, as shown by the decrease in average global PSQI scores from a baseline of 10.88 to 8.55 at the study endpoint. Memantine as augmentation therapy (mainly to SSRIs) was well tolerated. The most commonly reported adverse events included nausea 30% (*n* = 3), headaches 30% (*n* = 3), fatigue 20% (*n* = 2), and increased dream activity 20% (*n* = 2). One patient, 10%, also reported ankle edema.

## 4. Discussion

Glutamate is felt to play a role in the development of anxiety. Glutamate, an excitatory neurotransmitter, is often in balance with an inhibitory neurotransmitter, GABA. This GABA-glutamate balance (when GABA is low and Glutamate is normal to high) is also felt to play a role in the development of generalized or social anxiety disorders. Sometimes, GABA activity increasing sedative drugs, such as diazepam, are used to raise GABA and create a better balance between the stimulatory glutamate and inhibitory GABA. Given memantine's ability to lower glutamate activity, it may be able to also lower anxiety without the need for a sedative medication. Lowering glutamate this way may allow a patient's own GABA concentrations to be more effective in lowering generalized or social anxiety disorder symptoms.

Memantine may be an effective augmentation therapy in patients with anxiety who remain symptomatic despite adequate treatment with conventional antidepressant anxiolytics.

In regards to limitations, this was an open-label consecutive patient case series. As such, these data are open to referral and investigator bias. Future investigations would benefit from placebo-controlled clinical trials to further investigate the true benefits of memantine for the treatment of anxiety.

## Figures and Tables

**Table 1:** Demographic and clinical characteristics of patients.

	Mean	Range
Age	44 years	26–63 years

	Number	(%)

Gender		
Females	11	(74%)
Males	4	(26%)
Diagnosis		
Generalized anxiety disorder	15	(100%)
Comorbid social anxiety disorder	4	(26%)
Medications at time of enrollment		
Selective serotonin re-uptake inhibitor (SSRI)	15	(100%)
